# Comparing clinical outcomes of NOACs with warfarin on atrial fibrillation with Valvular heart diseases: a meta-analysis

**DOI:** 10.1186/s12872-019-1089-0

**Published:** 2019-05-15

**Authors:** Qiyu He, Chun-Yat Sze, Tin-Yau Shum, Guang Hao, Nga-Yin Belinda Wong, Tat-Hang Sin, Wei Wei, Sujian Xia

**Affiliations:** 1 Clinical Medicine of International School, Jinan University, Guangzhou, 510632 Guangdong China; 2 Department of Health Statistics, School of Medicine, Jinan University, No.601 Huangpudadao, Guangzhou, 510632 Guangdong China; 3 Department of Epidemiology, School of Medicine, Jinan University, Guangzhou, China

**Keywords:** Meta-analysis, NOAC, Warfarin, Atrial fibrillation, Valvular heart disease

## Abstract

**Background:**

Warfarin is the standard of care and NOAC (Novel oral anticoagulants) are a group of newer drugs for such purposes. NOAC has a generally better profile (Clear interaction, less side effect, require less monitoring). However, its efficacy on valvular atrial fibrillation remains unclear.

**Method:**

We researched literature articles from Embase, Cochrane and PubMed. Then we meta-analysed these six articles to assess pooled estimate of relative risk (RR) and 95% confidence intervals (Cl) using random-effects model for stroke, systemic embolic event, major bleeding and all-cause mortality. Heterogeneity across study was tested with Cochran’s Q Test and I^2^ Test. The bias of studies was first tested by examining the symmetry of Funnel Plot. Cochrane’s Collaboration Tool was also used to report any presented bias.

**Results:**

We collected 496 articles in total and finally we included six articles in our meta-analysis. For SSEE (Stroke, Systemic Embolic Event), the pooled relative risk showed a significantly better clinical outcome of NOAC (RR: 0.66; 95% CI: 0.46 to 0.95). However, there is no significant difference in major bleeding (RR: 0.714, 95% CI:0.46 to 1.11) and all-cause mortality (RR: 0.84, 95% CI: 0.58 to 1.21).

**Conclusion:**

Compared to Warfarin, NOAC is significantly more protective against the embolic event, but no significant difference in lowering risk of major bleeding, all-cause mortality or all aspects of post-TAVI (Trans-catheter aortic valve implantation).

**Electronic supplementary material:**

The online version of this article (10.1186/s12872-019-1089-0) contains supplementary material, which is available to authorized users.

## Background

Valvular heart disease (VHD) can increase the risk of stroke, atrial fibrillation (AF) and systemic embolic events (SSEE) [1], therefore, anticoagulants are commonly administrated for VHD patients. Vitamin K antagonist (VKA) i.e. warfarin was the standard of care and the only oral route available agent before the development of novel oral anticoagulants (NOACs). It can inhibit the synthesis of vitamin K-related coagulation factors, i.e. factor II, VII, IX, X and therefore can prevent thromboembolism. NOACs are newer drugs for treatment and prevention of thromboembolism. There are two major classes of NOACs, namely direct thrombin inhibitor which includes dabigatran; and factor Xa inhibitors which includes apixaban, edoxaban and rivaroxaban [2]. NOACs have more rapid pharmacokinetics effect, less side effects, and is more effective and don’t need to be monitored compared with warfarin, although they do not have antidotes, have limited usage in patients with renal impairment, and are more expensive than warfarin [3]. Although NOACs have a general better profile, their efficacy on valvular AF, especially for bioprosthetic valve, remain unclear [4]. Therefore, in patients with moderate or severe mitral stenosis or of a mechanical prosthetic heart valve, VKA is currently the only recommended oral anticoagulant for the prevention of SSEE [5].

However, recent studies implied that NOAC can also reduce the risk of SSEE in patients with valvular heart diseases. The RE-LY (Randomized Evaluation of Long Term Anticoagulation Therapy) trial with dabigatran [6], the ROCKET AF (Rivaroxaban Once Daily Oral Direct Factor Xa Inhibition Compared with Vitamin K Antagonism for Prevention of Stroke and Embolism Trial in Atrial Fibrillation) trial with rivaroxaban [7], the ARISTOTLE (Apixaban for Reduction in Stroke and Other Thromboembolic Events in Atrial Fibrillation) trial with apixaban [8, 9], and the ENGAGE AF–TIMI 48 (Effective Anti- coagulation with factor Xa Next Generation in Atrial Fibrillation–Thrombolysis In Myocardial Infarction 48) trial with edoxaban [10, 11] have included variable proportions of VHD patients. They showed that NOACs are not inferior to warfarin in patients with VHD for the main efficacy and safety outcomes. However, there are only a small portion of VHD patients enrolled in each trial. Also, the inclusion criteria of VHD patients in each trial are variable.

### Objective

Therefore, we would like to assess the outcome differences between NOACs and VKA in VHD patients with larger sample sizes by joint analysis of several different types of trials. We planned to focus on the VHD patients that have undergone valvular replacement surgery to evaluate the efficacy and safety outcomes. For this reason, we performed this meta-analysis of available comparative trials of NOACs versus VKA to compare the clinical outcomes of NOACs with VKA on management of valvular heart diseases.

## Methodology

### Protocol

This meta-analysis was planned and conducted under the statements for study design, data analysis and reporting of meta-analyses of RCT that are currently available and widely adopted. We adopted the protocol for systematic reviews and meta-analyses developed by Preferred Reporting Items for Systematic Reviews and Meta-Analyses (PRISMA) in order to improve the quality of the study [12].

### Eligibility criteria

For the type of studies, we included data from all published Controlled Intervention Studies that available for public access. We excluded all non-english studies, unfinished studies (before Phase III) and certain types of literature (including reviews, editorials, letters, notes, surveys, conference abstract). For the types of participants, we included patients with significant valvular heart disease (SVD). Significant valvular heart diseases are defined by follow characteristics [7] including: (1) Valve location or abnormality (including aortic stenosis, aortic regurgitation, mitral regurgitation and other), (2) Aetiologies including rheumatic, congenital, calcific/degenerative, post-infarction and/or ischaemic, other, unknown, no data, and (3) Prior cardiac valvular procedures, (including valvuloplasties and other cardiac valvular procedures). We excluded all studies using non-human subjects. For types of interventions and controls, we included treatment with only one of the following NOACs (Apixaban, Edoxaban, Dabigatran, Rivaroxaban), versus Warfarin, or its derivatives (e.g. Phenprocoumon) as the desired interventions and controls. We excluded studies that used combination therapy regimen other than only NOAC or only VKA (e.g. Combined regimen of NOAC with heparin or VKA with heparin, except anti-platelet).

We included specific efficacy outcome measures, namely Stroke, Systemic Embolic Event and all-cause mortality [7], and ISTH (International Society on Thrombosis and Haemostasis) classified major bleeding (including gastrointestinal bleeding and intracranial haemorrhage) are the targeted safety outcome. We excluded studies with unpublished result data from this meta-analysis. The eligibility criteria are documented in Table 1.

**Table 1:** Eligibility criteria applied in this meta-analysis

	Inclusion Criteria	Exclusion Criteria
Study type	All controlled intervention study	• Non-English study • Unfinished studies (Before Phase III) • Reviews, editorials, letters, notes, surveys, conference abstract
Participants	• SVD characterised by: 1. Primary and secondary valve abnormalities 2. Prior valvular procedures	Non-human subjects
Intervention	One of NOACs	Combined therapy with VKA
Control	Warfarin and its derivatives	Combined therapy with heparin
Outcome	• Efficacy: SSEE, Mortality • Safety: Major bleeding	Unpublished data

### Search strategy and information sources

We searched for all published Controlled Intervention Studies with results available for public access from the Cochrane, Embase and PubMed until 9 December 2018 under the following searching strategy listed in the Additional file 1. (Appendix I listed query for Cochrane and Appendix II listed query for Embase and PubMed) The study must include the quantitative outcome measures noted below (i.e. Efficacy outcome and Safety outcome) and reported in quantitative figures. Keywords used for search query includes, NOAC, Apixaban, Edoxaban, Dabigatran, Rivaroxaban, Warfarin, Vitamin K Antagonist and Valvular Heart Disease. We manually searched relevant journals recommended by the supervising professor that were not captured in the above databases.

### Study selection

**Fig. 1:**
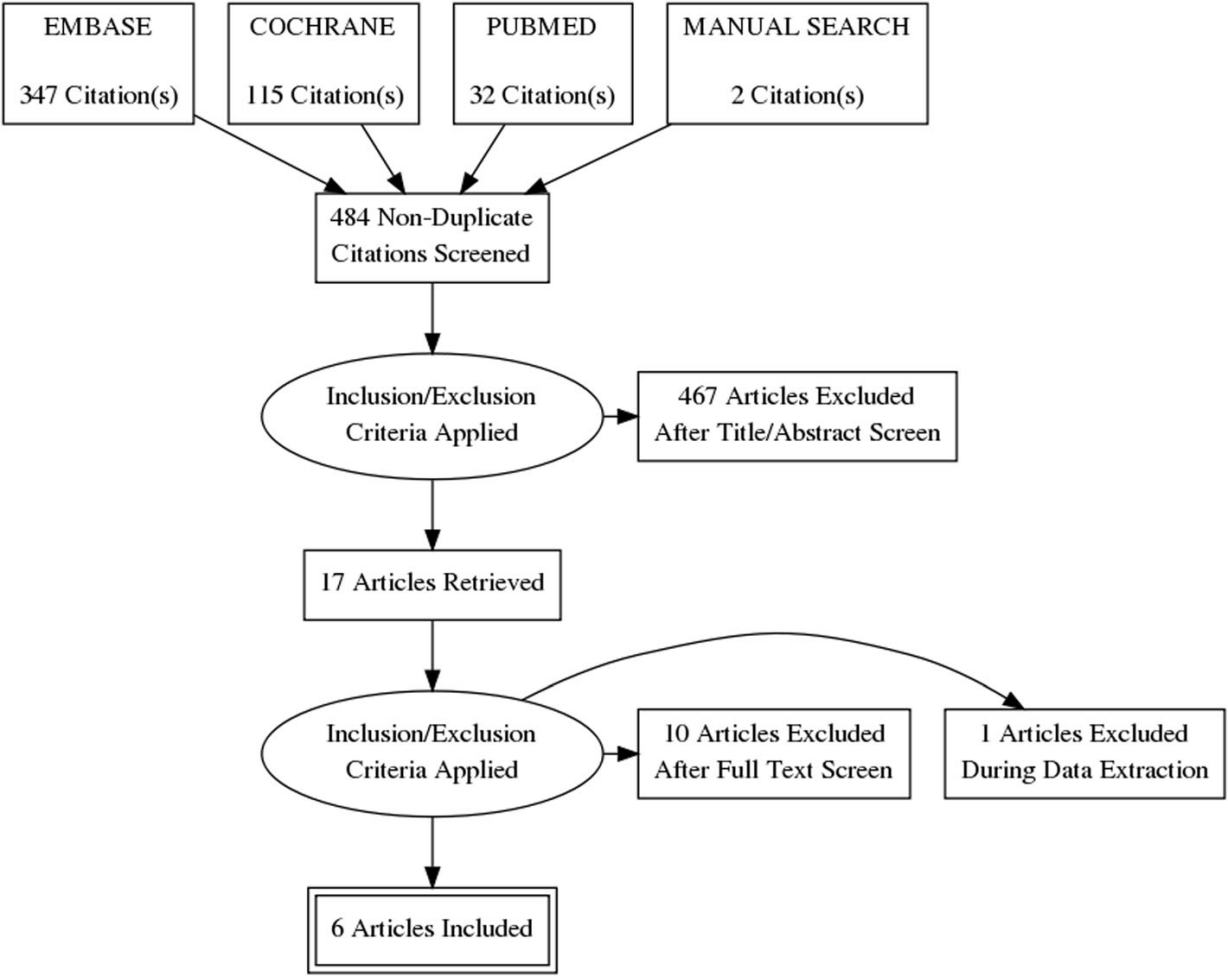
Graphical illustration of the selection process

Four authors independently participated and completed the initial title and abstract screening. We then retrieved full texts for the studies that were eligible. We resolved disagreements by meeting with all authors. The selection process is documented in a PRISMA (Preferred Reporting Items for Systematic Reviews and Meta-Analyses) flow diagram in Fig. 1.

### Data collection process

Four authors independently extracted data from all the eligible studies included. When data from a trial had been published more than once, it will be only extracted once. Disagreements between review authors were resolved by consensus after formal meetings.

### Data items

Data item for extraction of study characteristics were classified into two categories. First, regarding baseline characteristics which include first author, year of publication, study design, age, number of included individuals and disease status of participants in each group. Secondly, for outcome analysis, the characteristics include the followings: (1) Number of patients with and without SSEE in both groups, (2) Patient with and without major bleeding in both groups, (3) Deceased and survived patient in both groups respectively.

### Assessment of heterogeneity

These articles were analysed for statistical heterogeneity. We used Cochran’s Q test, I^2^ tests for heterogeneity among all studies [13]. We used the following criteria for heterogeneity: I^2^ > 50% for the presence of heterogeneity, and I^2^ > 70% high heterogeneity. Subgroup and sensitivity analyses were conducted.

### Synthesis of results and summary measures

Meta-analysis for a particular factor is performed using STATA version 14.0 MP. The principal summary measures are computed based on Relative Risk (RR) and 95% Confidence Interval (95% CI). Individual data were pooled using the Bootstrapped DerSimonian-Laird (BDL) random-effects model with 1000 repetition. The reason for using a BDL model is because of the data having significant heterogeneity (I^2^ > 50%), BDL random-effects model has better performance on small meta-analysis [13]. In a secondary analysis, we performed a subgroup analysis regarding the patient characteristics. We divided the 6 included trials into 2 group which were the group with VHD only and the group of post-TAVI patients. The model for pooling the RR and estimating 95% CI was the same. We performed Cochran’s Q test and I^2^ test to test for the heterogeneity.

### Risk of bias in individual studies

Two authors independently assessed the risk of bias in individual studies by using the Cochrane Collaboration’s tool, including selection, performance, detection, attribution and reporting. Moreover, Jackknife sensitivity analyses were performed for testing and estimating the impact of the individual study on the overall outcome by omitting one study at a time. Calculations were done by using a random-effects model.

### Risk of bias across studies

We aimed to minimise reporting bias by completing a comprehensive search for eligible studies while staying conscious of data duplication. A funnel plot was used for measuring publication bias (small study effect) [14]. If asymmetry is observed in the funnel plot, the Harbord’s modified test for small study effect will be used for analysis of the asymmetry, which is more suitable for analysis of dichotomous data [15]. The Cochrane collaboration tool was used to assess the risk of bias within individual study through the following items: selection bias, performance bias, attrition bias and reporting bias. The assessment was reported according to the method suggested by the Cochrane collaboration tool [13] and is listed in Table 2.

**Table 2:** Table reporting risk of bias in individual study

Bias	Judgement	Support
Article 1	ARISTOTLE [8, 9]	
Selection	Low	Quote: “we have randomized 18,206 patients with AF from over 1000 centers in about 40 countries. Eligible subjects were randomly assigned in a 1:1 ratio to receive either apixaban or warfarin …”
Performance	Low	Quote: “To maintain blinding, study medications are packaged using a double-dummy design. The 2 sets of tablets each subject receives are distinguishable by color and size, but active apixaban tablets match placebo apixaban tablets and active warfarin tablets match placebo warfarin tablets to ensure blinding of the patient and investigator.”
Detection	Low	
Attrition	Low	The reason of exclusion of patient was clearly stated
Reporting	Low	All pre-specified outcomes were reported as HR and 95%CI with respective number of patients
Article 2	ENGAGE AF-TIMI 48 [10, 11]	
Selection	Low	Quote: “Subjects are randomized through an interactive voice/ Web response system (IVRS) ... Approximately 20,500 patients will be enrolled with history of AF documented on an electrical recording within the past 12 months for whom anticoagulation is planned for the duration of the trial. Subjects are randomized 1:1:1 ... Randomization is stratified by CHADS_2_ score 2 to 3 versus 4 to 6 and drug clearance”
Performance	Low	Quote: “ENGAGE AF–TIMI 48 is a large, multinational, randomized (1:1:1), double-blind, double-dummy ... All subjects are dispensed 2 sets of study drug. The first set, edoxaban or matching placebo ... The second set, warfarin or matching placebo”
Detection	Low	Quote: “INR measurements are performed using a point-of-care device supplied to each study site. The INR results generated by the point-of-care device are masked by a “code number,” which is reported by the investigators to a central IVRS … ”
Attrition	Low	The reason of exclusion of patient was clearly stated
Reporting	Low	All pre-specified outcomes were reported as HR and 95%CI with respective number of patients
Article 3	RE-LY [6]	
Selection	Low	Quote: “The patients were randomized by a central randomization service, through an interactive voice response system (IVRS) located at the Coordinating Centre at Population Health Research Institute (PHRI) in Hamilton, Canada”
Performance	High	Open label
Detection	Low	Quote: “RE-LY is a phase 3, multicenter, prospective, open-label, randomized trial with blinded evaluation of all outcomes (PROBE design).”
Attrition	Low	The reason of exclusion of patient was clearly stated
Reporting	Low	All pre-specified outcomes were reported as HR and 95%CI with respective number of patients
Article 4	ROCKET-AF [7]	
Selection	Low	Quote: “Over 14,000 patients have been randomized in the ROCKET AF trial at 1100 sites across 45 countries. Patients are allocated to 1 of 2 study regimens: rivaroxaban or warfarin.”
Performance	Low	Quote: “A double-blind design was chosen to minimize bias in cointerventions and interpretation of clinical events. To maintain blinding in ROCKET AF, sham INR results are provided. A point-of-care coagulation testing device displays a code number that, when entered into the Interactive Voice Response System along with the subject’s study identification number, is decoded and generates either the subject’s real INR or a sham INR value, depending on the patient’s blinded treatment.”
Detection	Low	
Attrition	Low	The reason of exclusion of patient was clearly stated
Reporting	Low	All pre-specified outcomes were reported as HR and 95%CI with respective number of patients
Article 5	Seeger et. al., 2017 [16]	
Selection	High	The allocation of group is not randomised
Performance	High	The patient and the physician was not blinded
Detection	High	
Attrition	Low	The reason of exclusion of patient was clearly stated
Reporting	Low	All prespecified outcomes were reported as event rate with respective number of patients
Article 6	Geis et. al., 2018 [17]	
Selection	High	The allocation of group is not randomised and the study have retrospective design
Performance	High	The patient and the physician was not blinded
Detection	High	
Attrition	Low	The reason of exclusion of patient was clearly stated
Reporting	Low	All prespecified outcomes were reported as event rate with respective number of patients

The Cochrane Collaboration’s tool for assessing risk of bias

## Result

### Study selection

We retrieved 347 citations from EmBase, 115 citations from Cochrane, 32 citations from PubMed and included two citations from manual search. We exported those retrieved citations to EndNote X8 for further analysis. The software automatically identified 484 non-duplicated articles. According to the pre-specified selection criteria, we were able to exclude 467 articles based on the title and abstract of those studies. We performed a full-text screening on remained articles, which excluded ten more articles according to the selection criteria. Then, we extracted data from eight eligible articles. At the data extraction process, we excluded one more article as the article did not provide the desired data. Finally, we included six articles. Figure 1 showed the graphical illustration of the selection process according to the suggestion from PRISMA statement. Included studies covered an overall population of 14,120 patients who suffered from VHD and subjected to either NOAC or warfarin treatment.

### Study characteristics

The detail study characteristics are shown in Tables 3 and 4. Six selected articles are controlled clinical study. The patients included in the six trials are VHD patients, there were several different aetiology and pathology of VHD. For ARISTOTLE and ENGAGE AF-TIMI, bioprosthetic valve, mitral valve repair, native valve disease were included. For RE-LY, only native valve disease was included. Mitral valve repair and native valve disease are included in the trial of ROCKET AF. When it comes to Seeger et al. [16] and Geis et al. [17], only Post-TAVI of VHD was included in the study.

**Table 3:** Targeted drug used in each group with respective sample size and duration

Trial	Targeted Drug		Target sample size	Duration/ month
ARISTOTLE*	Intervention	Apixaban 5 mg BD	2438	21.8
(Avezum et al., 2015)	Control	Wafarin (INR 2–3)	2370	
ENGAGE AF-TIMI 48*	Intervention	Edoxaban 60 mg QD	1869	33.6
(De Caterina et al., 2017)	Control	Warfarin (INR 2–3)	955	
RE-LY	Intervention	Pradaxa 110/150 mg QD	2646	36
(Ezekowitz et al., 2016)	Control	Warfarin (INR 2–3)	652	
ROCKET AF*	Intervention	Rivaroxaban 20 mg QD	939	48
(Breithardt et al., 2016)	Control	Warfarin (INR 2–3)	1001	
Seeger et al.	Intervention	Apixaban	141	1, 12
−2017	Control	Phenprocoumon	131	
Geis et al.	Intervention	NOAC	154	6
−2018	Control	Warfarin (INR 2–3)	172

**Table 4:** Baseline characteristics of patients in each trial

Trial	Included VHD	Baseline Clinical Characteristics							
		Group	Age	CHADS2	HAS-BLED	HTN	CVA	DM	MI
ARISTOTLE*	Bioprosthetic valve	/	71 (64, 77)	2.2 ± 1.1	N/A	4102 (85.3)	905 (18.8)	1086 (22.6)	837 (17.4)
(Avezum et al., 2015)	Mitral valve repair								
	Native valve disease								
ENGAGE AF-TIMI 48* (De Caterina et al., 2017)	Bioprosthetic valve	HDER	71.8 ± 9.4	2.92 ± 1.0	2.55 ± 0.98	2629 (93.1)	668 (23.7)	908 (32.2)	1122 (39.8)
	Mitral valve repair	LDER							
	Native valve disease								
RE-LY	Native valve disease	Intervention: D110	74 (68, 78)	2 (1,3)	N/A	988 (76.5)	279 (21.6)	298 (23.1)	251 (19.4)
(Ezekowitz et al., 2016)		Intervention: D150	74 (67, 79)	2 (1,3)		1051 (77.7)	310 (22.9)	320 (23.7)	250 (18.5)
		Control	74 (68, 79)	2 (1,3)	N/A	1012 (77.5)	286 (21.9)	316 (24.2)	212 (16.2)
ROCKET AF*	Mitral valve repair	AS	78 (73, 82)	3.6 ± 0.9	3 ± 0.9	197 (92)	99 (46)	92 (43)	64 (30)
(Breithardt et al., 2016)	Native valve disease	MR/AR	74 (67, 79)	3.5 ± 1.0	2.8 ± 1.0	1542 (89)	829 (48)	690 (40)	404 (23)
Seeger et al.	Post-TAVI	/	81.3 ± 5.9	4.9 ± 1.2 (CHA2DS2VASc)	3.1 ± 1.1	N/A	35 (12.9)	32.4 (88)	19.5 (53)
−2017									
(Geis et al., 2018)	Post-TAVI	Intervention	83.1 ± 5.3	4.6 ± 1.2 (CHA2DS2VASc)	2.7 ± 0.8	147 (95)	24 (16)	47 (31)	80 (52)
		Control	83.0 ± 4.9	4.8 ± 1.3 (CHA2DS2VASc)	2.9 ± 0.8	158 (92)	25 (15)	57 (33)	88 (51)

### Synthesis of results

The meta-analysis aimed at comparing efficacy outcomes and safety outcomes of NOACs and Warfarin by conducting subgroup analysis, stratified by Valvular Heart Disease (VHD) and Transcatheter Aortic Valve Implantation (TAVI). The efficacy outcomes of interest for this meta-analysis were Stroke and Systemic Embolic Event, and all-cause death. Due to the data in ROCKET-AF RCT were not wholly documented in one article, data from the two articles were pooled for analysis.

The primary meta-analysis included 2438 patients received apixaban 5 mg twice a day and 2370 patients received warfarin titrated to INR 2.0 to 3.0 who were enrolled in ARISTOTLE trial; 1869 patients received edoxaban 60 mg once a day, and 955 patients received warfarin titrated to INR 2.0 to 3.0 who were enrolled in ENGAGE AF-TIMI 48 trial; 2646 patients received dabigatran 110 mg and 150 mg once a day, and 652 patients received warfarin titrated to INR 2.0 to 3.0 who were enrolled in RE-LY trial; 939 patients received rivaroxaban 20 mg once a day, and 1001 patients received warfarin titrated to INR 2.0 to 3.0 who were enrolled in ROCKET-AF trial; 141 patients received apixaban, and 131 patients received phenprocoumon who were enrolled in a trial conducted by Seeger et al. in 2017; 154 patients received NOAC, and 172 patients received warfarin titrated to INR 2.0 to 3.0 who were enrolled in a trial conducted by Geis et al. in 2018, the details of number of patient in each group is listed in Table 5. In a primary analysis, we applied BDL model to estimate pooled RR and its 95% CI. The rationale for using this model was that after testing for heterogeneity, we assumed that individual study true effects are distributed with variance τ^2^, around an “overall” true effect therefore a random-effect model was used to address for this issue [18].

**Table 5:** Original data used for the measurements and calculations

Study / Trial	With SEE		Without SEE		With Major Bleeding		Without Major Bleeding		All Cause Death		Survived	
	Intervention	Control	Intervention	Control	Intervention	Control	Intervention	Control	Intervention	Control	Intervention	Control
ARISTOTLE	64	89	2374	2281	99	119	2339	2251	222	215	2216	2155
ENGAGE TIMI 48	82	50	1787	905	99	89	1770	866	308	147	1561	808
RE-LY	77	98	2569	1206	209	264	2437	1040	226	244	2420	1060
ROCKET AF	38	50	901	951	253	240	686	761	100	112	839	889
Seeger	3	7	138	124	5	7	136	124	2	5	139	126
−2017												
Geis (2018)	4	2	150	170	11	11	143	161	12	11	142	161

### Risk of bias

**Fig. 2:**
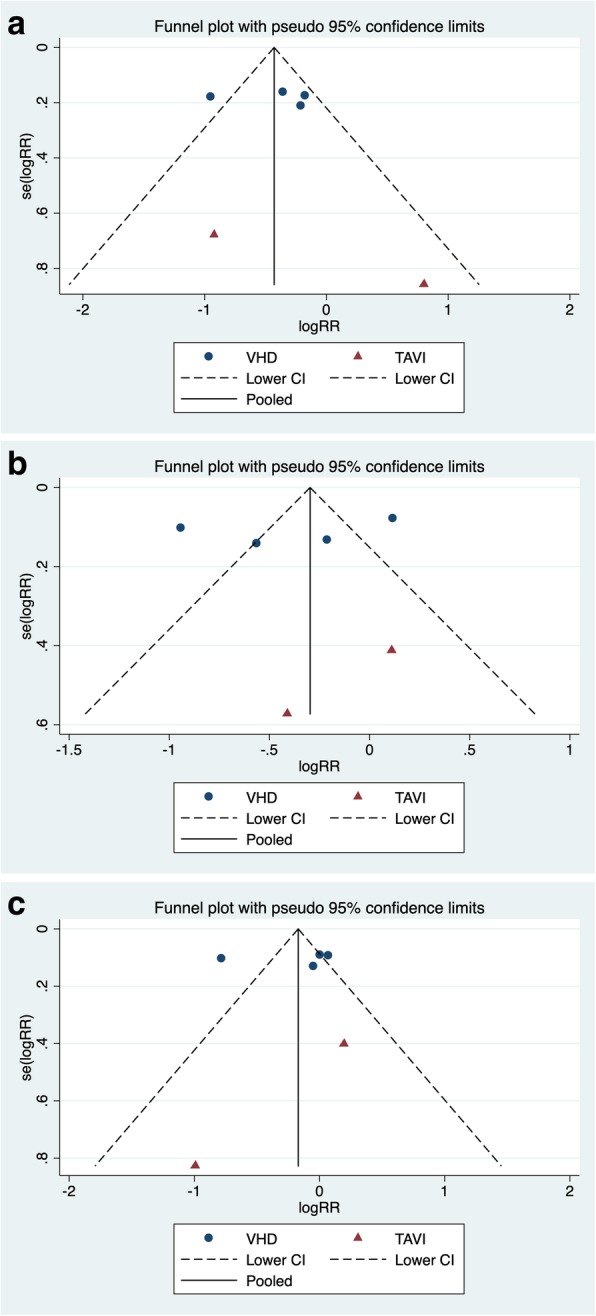
Funnel Plot for each individual outcome. 2a--SSEE; 2b--Major Bleeding; 2c--Mortality

**Fig. 3:**
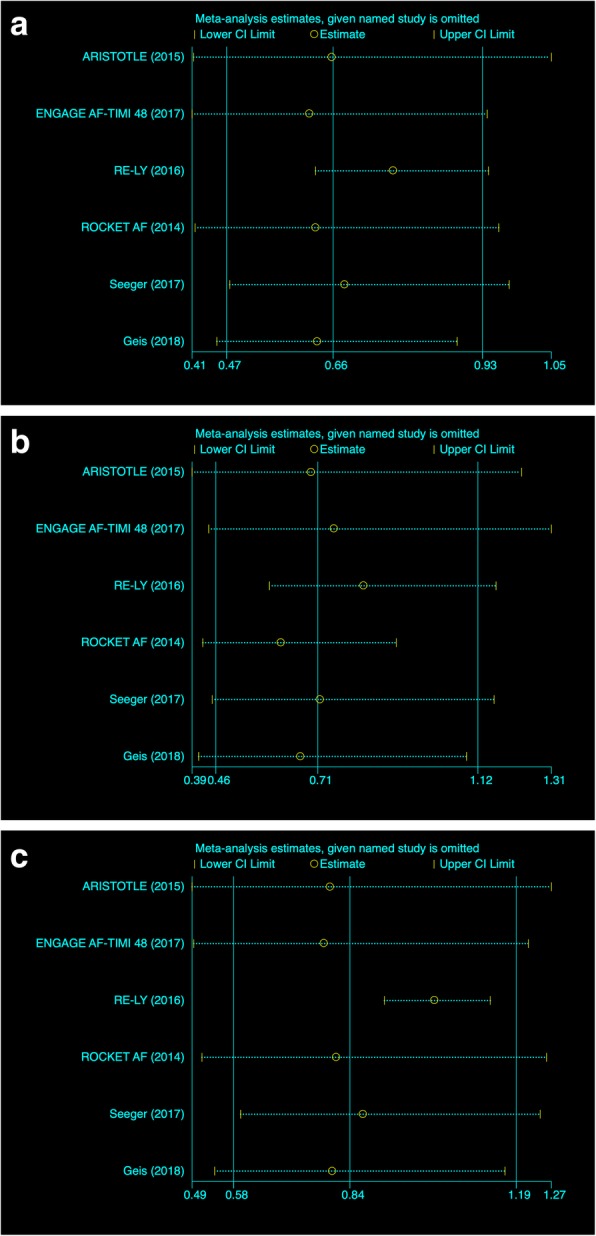
Graphical illustration of Jackknife Sensitivity test for each individual outcome. 3a--SSEE; 3b--Major Bleeding; 3c--Mortality

Visual inspection of the funnel plot (Fig. 2) for each outcome revealed no asymmetry which indicated that there was no publication bias [14]. The conclusion was later confirmed by Harbord’s modified test for small study effect. The funnel and the data of the Harbord’s test were listed in Tables 6, 7 and 8. The Jackknife sensitivity test verified that the RE-LY trial had a significant impact on the result of three outcomes. The visual representation is showed in Fig. 3.

**Table 6:** SSEE Analysis with Bootstrapped DerSimonian-Laird random-effects model calculated by STATA

STUDY	Overall			Subgroup: VHD			Subgroup: TAVI			Sensitivity Test		Harbord’s modified test
	RR	95%CI	Weight(%)	RR	95%CI	Weight(%)	RR	95%CI	Weight(%)	RR	95%CI	*p*-value
ARISTOTLE, 2015	0.699	0.510, 0.959	23.77	0.699	0.510, 0.959	26.35				0.661	0.415, 1.054	0.97
ENGAGE AF-TIMI 48, 2017	0.838	0.595, 1.181	22.99	0.838	0.595, 1.181	25.42				0.622	0.412, 0.939	
RE-LY, 2016	0.387	0.273, 0.548	22.85	0.387	0.273, 0.548	25.25				0.772	0.633, 0.941	
ROCKET AF, 2014	0.81	0.536, 1.224	20.95	0.81	0.536, 1.224	22.99				0.633	0.417, 0.960	
Seeger, 2017	0.398	0.105, 1.508	5.64				0.398	0.105, 1.508	54.56	0.685	0.479, 0.979	
Geis, 2018	2.234	0.415, 12.026	3.8				2.234	0.415, 12.026	45.44	0.636	0.456, 0.886	
Overall	0.665	0.468, 0.945	100	0.652	0.457, 0.930	100	0.872	0.159, 4.767	100	0.664	0.474, 0.931	
Heterogeneity Measures	Value	df	*p*-value	Value	df	*p*-value	Value	df	*p*-value			
Cochran’s Q	14.59	5	0.012	12.12	3	0.007	2.47	1	0.116			
Heterogeneity Measures	Value (95%CI)			Value (95%CI)			Value (95%CI)					
I2 (%)	62.7	9.41, 84.65		75.27	31.56, 91.07		60.52	0.00, 90.80				
H2	2.68	1.10, 6.51		4.04	1.46, 11.19		2.53	0.59, 10.87				
tau2 (BDL)	0.109			0.098			0.916

**Table 7:** Major bleeding Analysis with Bootstrapped DerSimonian-Laird random-effects model calculated by STATA

STUDY	Overall			Subgroup: VHD			Subgroup: TAVI			Sensitivity Test		Harbord’s modified test
	RR	95%CI	Weight(%)	RR	95%CI	Weight(%)	RR	95%CI	Weight(%)	RR	95%CI	*p*-Value
ARISTOTLE, 2015	0.809	0.623, 1.049	19.42	0.809	0.623, 1.049	24.61				0.698	0.395, 1.234	0.585
ENGAGE AF-TIMI 48, 2017	0.568	0.432, 0.749	19.27	0.568	0.432, 0.749	24.43				0.756	0.437, 1.31	
RE-LY, 2016	0.39	0.319, 0.477	19.99	0.39	0.319, 0.477	25.27				0.831	0.592, 1.169	
ROCKET AF, 2014	1.124	0.965, 1.309	20.35	1.124	0.965, 1.309	25.69				0.621	0.422, 0.915	
Seeger, 2017	0.664	0.216, 2.039	8.78				0.664	0.216, 2.039	34.06	0.72	0.446, 1.164	
Geis, 2018	1.117	0.498, 2.503	12.19				1.117	0.498, 2.503	65.94	0.671	0.411, 1.093	
Overall	0.714	0.461, 1.105	100	0.672	0.402, 1.122	100	0.935	0.486, 1.801	100	0.715	0.455, 1.122	
Heterogeneity Measures	Value	df	*p*-value	Value	df	*p*-value	Value	df	*p*-value			
Cochran’s Q	71.36	5	0	74.19	3	0	0.055	1	0.459			
Heterogeneity Measures	Value (95%CI)			Value (95%CI)			Value (95%CI)					
I2 (%)	91.12	83.43, 95.24		95.58	91.54, 97.69		0	0.00, 100.00				
H2	11.26	6.03, 21.00		22.62	11.82, 43.30		1	/				
tau2 (BDL)	0.238			0.261			0

**Table 8:** All-cause mortality analysis with Bootstrapped DerSimonian-Laird random-effect model calculated by STATA

STUDY	Overall			Subgroup: VHD			Subgroup: TAVI			Sensitivity Test		Harbord’s modified test
	RR	95%CI	Weight(%)	RR	95%CI	Weight(%)	RR	95%CI	Weight(%)	RR	95%CI	*p*-Value
ARISTOTLE, 2015	1.004	0.839, 1.200	21.54	1.004	0.839, 1.200	25.42				0.792	0.494, 1.271	0.674
ENGAGE AF-TIMI 48, 2017	1.071	0.894, 1.282	21.52	1.071	0.894, 1.282	25.4				0.779	0.498, 1.221	
RE-LY, 2016	0.456	0.373, 0.559	21.23	0.456	0.373, 0.559	25.05				1.018	0.910, 1.139	
ROCKET AF, 2014	0.952	0.738, 1.228	20.46	0.952	0.738, 1.228	24.14				0.806	0.515, 1.26	
Seeger, 2017	0.372	0.073, 1.883	4.18				0.372	0.073, 1.883	34.42	0.864	0.599, 1.247	
Geis, 2018	1.218	0.554, 2.681	11.08				1.218	0.554, 2.681	65.58	0.797	0.543, 1.171	
Overall	0.835	0.578, 1.205	100	0.827	0.556, 1.229	100	0.811	0.244, 2.692	100	0.835	0.584, 1.195	
Heterogeneity Measures	Value	df	*p*-value	Value	df	*p*-value	Value	df	*p*-value			
Cochran’s Q	48.87	5	0	46.38	3	0	1.65	1	0.2			
Heterogeneity Measures	Value (95%CI)			Value (95%CI)			Value (95%CI)					
I2 (%)	87.89	76.10, 93.86		93.48	86.52, 96.52		49.35	0.00, 100.00				
H2	8.26	4.18, 16.30		15.35	7.42, 31.76		1.97	/				
tau2 (BDL)	0.155			0.153			0.408

### Study result

The result showed that there was a statistically significant heterogeneity across all three focused outcomes (I^2^ > 50%, Cochran’s Q test *P*-value < 0.05), the value for each test are listed in Tables 6, 7 and 8.

**Fig. 4:**
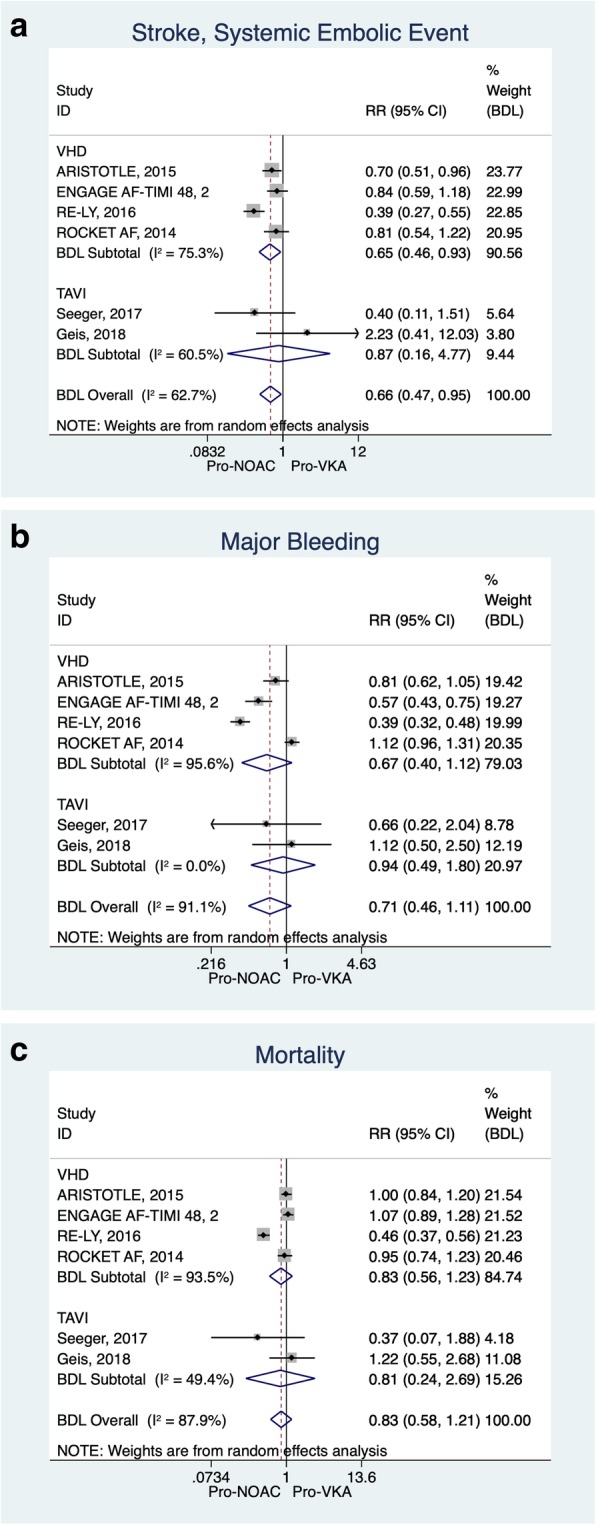
Forest Plot for each individual outcome. There are 4a, 4b and 4c multi-panel figures included

The main characteristics of patients in the included articles were shown in Tables 3, 4 and the respective medication received with dosage and frequency, the targeted INR was reported for patients receiving warfarin. Generally, regarding the clinical outcomes, SSEE rate in NOAC group was significantly lower than that of warfarin group (RR: 0.665; 95% CI: 0.468 to 0.945). The protective effect of NOACs was more substantial in the RE-LY trial (RR: 0.387; 95% CI: 0.273 to 0.548). However, Geis revealed a better performance of warfarin on valvular heart diseases (RR: 2.234; 95% CI: 0.415 to 12.026). Heterogeneity was proven to exist among the studies (I^2^ = 62.70%). For ISTH Major Bleeding, only ENGAGE AF-TIMI 48 and RE-LY trials showed the protective effect of NOACs are more significant than that in warfarin, and the ENGAGE AF-TIMI 48 trial showed higher RR than that in RE-LY (RR: 0.390; 95% CI: 0.319, 0.477). In contrast, other four trials showed no significant difference in effect of NOACs compared with the warfarin group. The overall result in Major Bleeding showed there was no significant effect favouring NOACs or VKA (RR: 0.714; 95% CI: 0.461, 1.105). The heterogeneity I^2^ test was performed and showed that heterogeneity across studies have a great chance of affect the pooled RR (I^2^ = 87.89%). For All-Cause-Mortality, only RE-LY trial showed the more significant protective effect of NOACs compared with warfarin (RR: 0.456; 95% CI: 0.373, 0.559), while other five trials showed no significant difference of the protective effect of NOACs compared with warfarin. In essence, the overall results of All-Cause-Mortality showed there was no significant difference in effect of NOACs compared with warfarin (RR: 0.835; 95% CI: 0.578, 1.205). The detailed data and corresponding forest plot is showed in Tables 6, 7 and 8 and Fig. 4.

Moreover, we also performed subgroup analysis of VHD-only group and post-TAVI group, to analysis more details about the effect of NOACs and warfarin under different patient characteristics. For subgroup of VHD, ARISTOTLE trial (RR: 0.699; 95% CI: 0.510, 0.959) and RE-LY trial (RR: 0.387; 95% CI: 0.273, 0.548) showed positive protective effect of NOACs than warfarin while ENGAGE AF-TIMI (RR: 0.838; 95% CI: 0.595, 1.181) and ROCKET-AF (RR: 0.810, 95% CI: 0.536, 1.224) showed negative results in event of SSEE. Also, the overall results (RR: 0.652; 95% CI: 0.457, 0.930) showed that there was a protective effect of NOACs compared with warfarin in SSEE event. For ISTH Major Bleeding in subgroup analysis of VHD, ENGAGE AF-TIMI 48 trial (RR: 0.568; 95% CI: 0.432, 0.749) and RE-LY trial (RR: 0.390; 95% CI: 0.319, 0.477) indicated NOAC was more protective than warfarin, whereas the overall results (RR: 0.672; 95% CI: 0.402, 1.122) showed negative protective results of NOACs and warfarin. For All-Cause-Mortality in the subgroup analysis of VHD, RE-LY trial indicated that there was a positive protective effect of NOACs compared with warfarin (RR: 0.456; 95% CI: 0.373, 0.559). However, the overall results of four trials included in subgroup analysis demonstrated no extra protective effect of NOAC (RR: 0.827, 95% CI: 0.556, 1.229). In the subgroup analysis of TAVI, Seeger trial indicated a stronger protective effect of NOACs than that in warfarin (RR: 0.398; 95% CI: 0.105, 1.508) but no effective protection in overall results (RR: 0.872; 95% CI: 0.159, 4.767). When it comes to ISTH Major Bleeding in subgroup TAVI, no statistical significance were portrayed between two trials (Overall: RR: 0.935; 95% CI: 0.486, 1.801). For All-Cause-Mortality in the subgroup of TAVI, both trials showed statistical insignificance between NOACs and warfarin (RR: 0.811; 95% CI: 0.244, 2.692).

The subgroup analysis of VHD showed the same result as overall analysis with respective RR 0.652 and 0.665, 95% CI: 0.457, 0.930 vs 95% CI: 0.468, 0.945, which the value of each dataset were similar and indicated almost the same result between overall and subgroup analysis. However, there are several factors can influence the consequence: the change of population of each trial, different methodology, and different baseline characteristics of patients can lead to the variation of the analysis result.

## Discussion

### Protecting effect on VHD

From our analysis, the superiority of NOACs over warfarin in reducing the rate of stroke and systemic embolic events is consistent with previous studies [8, 10, 19, 20]. This is an encouraging result towards its application on AF management with VHD. However, NOAC failed to show its superiority in reducing major bleeding and all-cause mortality rate. A previous pharmacological study done on Dabigatran showed that gastrointestinal bleeding risk increased with age [21] while Breithardt et al. proposed increased bleeding event in patients with aortic stenosis were associated with type 2A von Willebrand Syndrome. Despite adequate evidence suggesting NOAC-related bleeding adverse effect, the exact cause needs further investigation, and it may relate to the clinical characteristics of participants. From our analysis, no significant hazardous bleeding of NOAC is documented when compared to conventional oral anticoagulation. All-cause mortality of NOAC in VHD patients has not yet proven to have advantages over warfarin. Expectedly, the mortality rate of VHD is mainly determined by multiple variables. Provided that NOACs significantly reduced the mortality rate from acute embolic events, the complications secondary to pre-existed VHD including heart failure, ventricular arrhythmia, pulmonary oedema, pneumonia may not benefit from NOACs.

According to our analysis of various trials, RE-LY Trial demonstrated the trend that NOAC exhibited the most significant benefit when compared with other selected trials. At the same time, the RE-LY trial showed NOAC superiority in reducing SSEE, major bleeding and all-cause mortality. The explanation was unknown, but several reasons may be responsible. Focusing on the study design, the main characteristic of the RE-LY Trial was the selection of dabigatran. Not similar to other NOAC, dabigatran acts as a direct thrombin inhibitor. However, whether this mechanism exerts an additional protective effect on VHD required further investigations. However, some patients co-administered with amiodarone may enhance the effect of dabigatran [3]. The drug interaction may be one of the associated factors, though it failed to give us a satisfactory explanation. Assume that drug interaction is significant, the major bleeding rate should be aggravated whereas the result showed a contradiction. Apart from the intervention drug, the inclusion criteria also differed from other trials. Only native heart diseases such as aortic stenosis, aortic regurgitation and mitral regurgitation while moderate-to-severe mitral stenosis is excluded. As RE-LY trial only recruited haemodynamically stable subjects, less embolic events, bleeding as well as all-cause mortality seemed reasonable. The last variable regarding methodology was the inclusion criteria. The inclusion criteria of this meta-analysis did not investigate the factor of dosage. Thus different dosage of dabigatran group was recruited as a single intervention group. This factor makes the RE-LY trial having the largest sample sizes in the intervention group and the smallest sample size in the control group. In RE-LY trial, higher dose dabigatran was proven to be more beneficial.

On the contrary, an indirect comparison of dabigatran and other NOAC was performed in this study. Besides, RE-LY Trial is an open-label trial, which may exert some influence in the intervention group. Nonetheless, more studies were required to confirm the superiority of dabigatran.

### Role of NOAC in valve surgery

Since NOAC has been introduced to anti-coagulating therapy, its role in valvular surgery has been investigated. RE-ALIGN trial has explored the efficacy of dabigatran in patients with mechanical valves. However, major bleeding, as well as embolic events, were significantly higher than the control group [22], and hence, the trial was terminated prematurely in phase 2 [23] and subjects implanted with mechanical valve were excluded in most of NOAC-related trials [8, 10, 19, 20]. Warfarin remained the standard of care in mechanical valve replacement [24].

Although NOAC has been proven to be harmful on mechanical valve, its effect on bioprosthetic valve has been questioned. Among the selected trials, ENGAGE AF-TIMI 48 and ARISTOTLE included bioprosthetic valve replacement as eligible candidates, but analysis of NOAC effect on valve surgery has not been clearly stated [8, 10]. Thus, additional analysis of similar trials targeting TAVI [16, 17] was performed to explore the effectiveness of NOAC on TAVI. SSEE, major bleeding and all-cause mortality were evaluated in the TAVI sub-group. Different from non-surgical subjects, NOAC showed no superiority over warfarin in reducing SSEE (RR: 0.87; 95% CI: 0.16 to 4.77), major bleeding (RR: 0.94; 95% CI: 0.49 to 1.80) and all-cause mortality (RR: 0.81; 95% CI: 0.24 to 2.69).

The exact mechanism of corresponding findings was unknown, but some researchers [25, 26] reported there was microthrombi formation on bioprosthetic valve after NOAC administration. Sun, Davidson, Lamy & Eikelboom et al. (2009) [24] proposed that thrombogenicity may result from altered blood flow, iatrogenic tissue injury and exposed surgical material (e.g. suture) and post-TAVI thrombogenicity would decrease within 3 months after completion of bioprosthetic valve endothelisation. Theoretically, by their theory, anti-coagulation were optimal within this period, which may explain the better protective effect in Seeger et al. (2018) trial (RR: 0.4; 95% CI: 0.11 to 1.51) with shorter 1-month follow-up. However, only this reason may not account for the phenomenon. Focusing on the methodology of both trials [16, 17], Seeger selected the participants receiving post-operative 4-week anti-platelet therapy while Geis only recruited patients with NOAC monotherapy. Whether anti-platelet agents participated in lowering risk in Seeger et al. (2018) [16] studies was unknown, but Nishimura et al. (2014) [5] supported the anti-platelet prophylaxis in bioprosthetic valve management. Meanwhile, “Global Study Comparing a Rivaroxaban-based Antithrombotic Strategy to an Antiplatelet-based Strategy After Transcatheter Aortic Valve Replacement to Optimize Clinical Outcomes” (GALILEO) trial, another randomised control trial, is being conducted to investigate the protective effect of the antiplatelet agents in TAVI [27]. Owing to small sample sizes together with limited large-scale trials, the only brief conclusion about non-superiority of NOAC on TAVI was drawn from this meta-analysis. Two large RCTs were conducting to explore the efficacy of NOAC towards TAVI [17]. “Anti-Thrombotic Strategy After Trans-Aortic Valve Implantation for Aortic Stenosis” (ATLANTIS) aimed at comparing apixaban and warfarin on TAVI for AS while “Edoxaban Compared to Standard Care After Heart Valve Replacement Using a Catheter in Patients With Atrial Fibrillation” (ENVISAGE-TAVI) compared another factor Xa inhibitor, Edoxaban, with standard of care in TAVI patient. With these two studies, the significance and clinical application of NOAC in TAVI would be expected.

### Clinical implications

With the results from this meta-analysis, NOAC was shown to be more beneficial in lowering the chance of stroke and systemic embolic event in VHD except for post-TAVI patients. Till now, warfarin remained to be the main anti-coagulant for VHD and valve surgery [5, 24]. Warfarin was the only choice in the mechanical valve, and adjuvant anti-platelet is necessary for the bioprosthetic valve [24]. There are more ongoing studies to assess the effectiveness of NOAC in VHD or bioprosthetic valve. Despite limited evidence proving the efficacy, NOAC can be considered in some VHD patients as long as it is not absolutely contraindicated. The physician could make the clinical decision by balancing the risk of ischaemic and bleeding. CHA2DS2VASc Score was a commonly used tool to evaluate the possible ischaemic risk, and HAS-BLED or HEMORR2HAGES were two commonly adopted tools to estimate the bleeding risk [28]. Careful evaluation should be made in VHD patients before the NOAC prescription given that insufficient robust evidence-based RCT proved its role [17].

### Limitations

No universal consensus has been made regarding the definition of valvular heart diseases until now, though 2014 AHA/ACC/HRS guideline stated the definition of non-valvular diseases as the absence of rheumatic mitral stenosis, mechanical/Bioprosthetic heart valve or mitral valve repair [19]. As stated in the study characteristics (See Tables 3 and 4), different exclusion criteria of VHD and study design among various clinical trial is noted, which may affect the consistency as well as analysis of intervention on VHD. Even the samples shared the same diagnosis. Its determination was mainly dependent on the individual clinical judgement of local centre [8]. Though Dabigatran is categorised as NOAC, its direct thrombin inhibiting mechanism is different from Factor Xa inhibition of “Xaban”, which provided uncertainty of the intervention effect.

On the other hand, the choice of valve materials of patients undergoing valve surgery (i.e. Bioprosthetic valve types) with anticoagulation may have a variable effect on the prognosis. VHD is a broad category of different cardiac conditions that may have entirely different aetiologies, pathogenesis, treatments and prognosis. There is insufficient literature focusing on the sub-groups analysis [20].

On top of that, limited classification or access of VHD sub-groups in both intervention and control group is one of the possible barriers of further data analysis [8, 10]. Due to limited eligible sampling size, this study only included valve surgery without sub-dividing valve material and it may mask the effect of certain valve materials. Nevertheless, there is an ongoing large-scale ATLANTIS and ENVISAGE-TAVI trial [17] investigating the NOAC effect on TAVI, which may provide more information in the future. Further specific investigations targeting sub-groups of VHD, particularly native heart diseases are still necessary.

## Conclusion

Several studies investigating NOAC and Warfarin on AF with VHD were selected to perform a meta-analysis. NOAC was shown to be superior to warfarin in reducing stroke and systemic embolic event in VHD but not major bleeding and all-cause mortality. Surgical sub-group regarding bioprosthetic TAVI were analysed among those studies revealed NOAC had no advantages over the warfarin group. Further trials about the VHD sub-groups and TAVI were necessary.

The available evidences were not enough to provide a recommended agent of choice. We advise physicians choose the agent based on a case to case, individualized analysis of the valvular pathology, functional status of the patient and the socioeconomic status to provide a management plan that is most suitable to the patient.

## Additional file

Additional file 1: Searching query. Searching query of Cochrane, EmBase and PubMed (DOCX 15 kb)

## Abbreviations

BDL: Bootstrapped DerSimonian-Laird
ISTH: International society on thrombosis and Haemostasis
NOAC: Novel Oral anti-coagulant
SSEE: Stroke, systemic embolic event
SVD: Significant valve disease
TAVI: Transcatheter aortic valve implantation
VHD: Valvular heart disease
VKA: Vitamin K antagonist
