# Supplementary material for: Comparing clinical outcomes of NOACs with warfarin on atrial fibrillation with Valvular heart diseases: a meta-analysis
Source: BMC Cardiovasc Disord. 2019 May 15;19:113. doi: 10.1186/s12872-019-1089-0 (PMC6521383; doi:10.1186/s12872-019-1089-0)
Supplement: Supplementary file 1 — Searching query. Searching query of Cochrane, EmBase and PubMed (DOCX 15 kb) [file 12872_2019_1089_MOESM1_ESM.docx]

# Appendix I Cochrane Search Query

ID Search

#1 MeSH descriptor: [Factor Xa Inhibitors] explode all trees

#2 (Rivaroxaban or Xarelto):ti,ab,kw

#3 (Apixiaban or Eliquis):ti,ab,kw

#4 (Edoxaban or Lixiana):ti,ab,kw

#5 (NOAC or Novel Oral Anti-coagulant or Novel Anticoagulant):ti,ab,kw

#6 (Non vitamin K anti-coagulant):ti,ab,kw

#7 MeSH descriptor: [Dabigatran] explode all trees

#8 #1 OR #2 OR #3 OR #4 OR #5 OR #6 OR #7

#9 MeSH descriptor: [Warfarin] explode all trees

#10 (Warfarin or couma*):ti,ab,kw

#11 (Anti-coagulant*):ti,ab,kw

#12 ("anticoagulant" or conventional anti-coagul*):ti,ab,kw

#13 (Vitamin K antagon* or VitaminK antagon*):ti,ab,kw

#14 #9 OR #10 OR #11 OR #12 OR #13

#15 MeSH descriptor: [Heart Valve Prosthesis Implantation] explode all trees

#16 MeSH descriptor: [Heart Valve Diseases] explode all trees

#17 (Heart, valv* or Prosthe* valv*):ti,ab,kw

#18 (Valvoplasty or valv* surgery or surgical valv*):ti,ab,kw

#19 (Artificial valv* or tissue valve or bioprosthetic):ti,ab,kw

#20 ("valve replacement"):ti,ab,kw

#21 ("Mitral Stenosis" or "Mitral Regurgitation"):ti,ab,kw

#22 ("Aortic Stenosis" or "Aortic Regurgitation"):ti,ab,kw

#23 #15 OR #16 OR #17 OR #18 OR #19 OR #20 OR #21 OR #22

#24 #8 AND #14 AND #23

# Appendix II EmBase and PubMed Search Query

("valvular heart disease" OR "cardiac valve defect" OR "cardiac valve disease" OR "cardial valve disease" OR "heart valve abnormalities" OR "heart valve abnormality" OR "heart valve defect" OR "heart valve degeneration" OR "heart valve disease" OR "heart valve diseases" OR "heart valve lesion" OR "heart valvular disease" OR "valve disease, heart" OR "valvular heart disease" OR "valvulopathies" OR "valvulopathy" OR "vitium cordis")

AND

("apixaban" OR "1 (4 methoxyphenyl) 7 oxo 6 [4 (2 oxopiperidin 1 yl) phenyl] 4, 5, 6, 7 tetrahydro 1h pyrazolo [3, 4 c] pyridine 3 carboxamide" OR "4, 5, 6, 7 tetrahydro 1 (4 methoxyphenyl) 7 oxo 6 [4 (2 oxo 1 piperidinyl) phenyl] 1h pyrazolo [3, 4 c] pyridine 3 carboxamide" OR "apixaban" OR "bms 562247" OR "bms 562247 01" OR "bms 562247-01" OR "bms562247" OR "bms562247 01" OR "bms562247-01" OR "eliques" OR "eliquis" OR "edoxaban" OR "du 176" OR "du 176b" OR "du176" OR "du176b" OR "edoxaban" OR "edoxaban tosilate" OR "edoxaban tosilate hydrate" OR "edoxaban tosylate" OR "edoxaban tosylate hydrate" OR "endoxaban" OR "lixiana" OR "n (5 chloro 2 pyridinyl) n` [4 (n, n dimethylcarbamoyl) 2 (5 methyl 4, 5, 6, 7 tetrahydrothiazolo [5, 4 c] pyridine 2 carboxamido) cyclohexyl] oxamide" OR "roteas" OR "savaysa" OR "rivaroxaban"/exp OR "5 chloro n [ [2 oxo 3 [4 (3 oxomorpholin 4 yl) phenyl] 1, 3 oxazolidin 5 yl] methyl] thiophene 2 carboxamide" OR "5 chloro n [ [2 oxo 3 [4 (3 oxomorpholin 4 yl) phenyl] oxazolidin 5 yl] methyl] thiophene 2 carboxamide" OR "5 chloro n [ [2 oxo 3 [4 (3 oxomorpholino) phenyl] 5 oxazolidinyl] methyl] 2 thiophenecarboxamide" OR "bay 59 7939" OR "bay 59-7939" OR "bay 597939" OR "bay59 7939" OR "bay59-7939" OR "bay597939" OR "rivaroxaban" OR "xarelto" OR "dabigatran" OR "bibr 953" OR "bibr953" OR "dabigatran" OR "n [ [2 [ (4 amidinoanilino) methyl] 1 methyl 5 benzimidazolyl] carbonyl] n (2 pyridyl) beta alanine" OR "n [ [2 [ [[4 (aminoiminomethyl) phenyl] amino] methyl] 1 methyl 1h benzimidazol 5 yl] carbonyl] n (2 pyridyl) beta alanine")

AND

("warfarin" OR "1 (4` hydroxy 3` coumarinyl) 1 phenyl 3 butanone" OR "3 (alpha acetonylbenzyl) 4 hydroxycoumarin" OR "3 acetonylbenzonyl 4 hydroxy coumarinedimethylaminoethanol" OR "3 alpha phenyl beta acetylethyl 4 hydroxycoumarin" OR "acetonylbenzylhydroxycoumarin" OR "adoisine" OR "aldocumar" OR "alpha acetonylbenzyl 4 hydroxycoumarin dimethylaminoethanol" OR "antrombin k" OR "athrombin" OR "athrombin k" OR "athrombin-k" OR "athrombine k" OR "athrombinek" OR "befarin" OR "carﬁn" OR "circuvit" OR "compound 42" OR "coumadan" OR "coumadan sodico" OR "coumadin" OR "coumadin sodium" OR "coumadine" OR "coumafene" OR "coumaphene" OR "d warfarin" OR "dagonal" OR "dextro warfarin" OR "farin" OR "jantoven" OR "kumatox" OR "l warfarin" OR "levo warfarin" OR "maforan" OR "marevan" OR "orfarin" OR "panwarfarin" OR "panwarﬁn" OR "potassium warfarin" OR "prothromadin" OR "r warfarin" OR "simarc-2" OR "sodium warfarin" OR "sodium warfarinum" OR "sofarin" OR "tintorane" OR "uniwarfin" OR "wafarin" OR "waran" OR "warf compound 42" OR "warfar" OR "warfarin" OR "warfarin 2 (dimethylamino) ethanol" OR "warfarin potassium" OR "warfarin sodium" OR "warfarine" OR "warfarinum sodium" OR "warfil 5" OR "warﬁlone" OR "warnerin")
